# Assessment of global DNA methylation in peripheral blood cell subpopulations of early rheumatoid arthritis before and after methotrexate

**DOI:** 10.1186/s13075-015-0748-5

**Published:** 2015-08-29

**Authors:** María C. de Andres, Eva Perez-Pampin, Manuel Calaza, Francisco J. Santaclara, Ignacio Ortea, Juan J. Gomez-Reino, Antonio Gonzalez

**Affiliations:** Laboratorio de Investigacion 10 and Rheumatology Unit, Instituto de Investigación Sanitaria-Hospital Clínico Universitario de Santiago, Travesia de Choupana, s/n, 15706 Santiago de Compostela, Spain; Department of Medicine, University of Santiago de Compostela, Rúa de San Francisco, s/n, 15782 Santiago de Compostela, Spain

## Abstract

**Introduction:**

DNA methylation is an epigenetic mechanism regulating gene expression that has been insufficiently studied in the blood of rheumatoid arthritis (RA) patients, as only T cells and total peripheral blood mononuclear cells (PBMCs) from patients with established RA have been studied and with conflicting results.

**Method:**

Five major blood cell subpopulations: T, B and NK cells, monocytes, and polymorphonuclear leukocytes, were isolated from 19 early RA patients and 17 healthy controls. Patient samples were taken before and 1 month after the start of treatment with methotrexate (MTX). Analysis included DNA methylation with high-performance liquid chromatography-electrospray ionization-tandem mass spectrometry-selected reaction monitoring (HPLC-ESI-MS/MS-SRM) and expression levels of seven methylation-specific enzymes by quantitative polymerase chain reaction (qPCR).

**Results:**

Disease-modifying anti-rheumatic drug (DMARD)-naïve early RA patients showed global DNA hypomethylation in T cells and monocytes, together with a lower expression of DNA methyltrasnferase 1 (*DNMT1*), the maintenance DNA methyltransferase, which was also decreased in B cells. Furthermore, significantly increased expression of ten-eleven translocation1 (*TET1*), *TET2* and *TET3*, enzymes involved in demethylation, was found in monocytes and of *TET2* in T cells. There was also modest decreased expression of *DNMT3A* in B cells and of growth arrest and DNA-damage-inducible protein 45A (*GADD45A*) in T and B cells. Treatment with MTX reverted hypomethylation in T cells and monocytes, which were no longer different from controls, and increased global methylation in B cells. In addition, *DNMT1* and *DNMT3A* showed a trend to reversion of their decreased expression.

**Conclusions:**

Our results confirm global DNA hypomethylation in patients with RA with specificity for some blood cell subpopulations and their reversal with methotrexate treatment. These changes are accompanied by parallel changes in the levels of enzymes involved in methylation, suggesting the possibility of regulation at this level.

**Electronic supplementary material:**

The online version of this article (doi:10.1186/s13075-015-0748-5) contains supplementary material, which is available to authorized users.

## Introduction

Epigenetics has become an area of interest for the study of rheumatoid arthritis (RA) [1]. It refers to stable but reversible changes in gene expression that are heritable through cell divisions but do not involve DNA variants. They maintain the cellular phenotypes acquired during development and differentiation, and reflect physiological changes and environmental stress. This sort of cellular memory is used to promote adaptive phenotypic changes that result in increased fitness, but when aberrant they could promote or perpetuate a disease status. Epigenetic mechanisms include DNA methylation, histone modifications, microRNA, other non-coding RNA and nucleosome positioning. Current interest is based on findings indicating that epigenetic changes can become biomarkers to differentiate patients from healthy controls and to separate patient subgroups on prognosis, or on response to treatment, as well as to serve as new targets for treatment [2]. In addition, changes in DNA methylation of blood cells have been characterized as mediators of genetic risk in RA and are of interest to understand disease pathogenesis [3].

DNA methylation is the most widely studied and well-characterized epigenetic change [4]. It happens mainly as 5-methylycytosine (5mC) at C–phosphate–G dinucleotides (CpG) by the enzymatic transfer of a methyl group from S-adenosyl-L-methionine (SAM). In the bulk genome CpG are rare and highly methylated, but in clusters of CpG dinucleotides called CpG islands they are usually non-methylated. When CpG islands at gene promoters are methylated they are associated with long-term silencing of gene expression. In contrast, variable and tissue-specific methylation takes place with preference outside CpG islands [4]. Maintenance of the methylated status through mitosis requires a specific DNA methyltransferase (DNMT), DNMT1, which recognizes hemimethylated DNA sequences and methylates the new DNA strand. Two other enzymes of the same family, DNMT3A and DNMT3B, have been characterized as required for de novo methylation during development or in response to environmental stimuli or other stress. Active demethylation depends on the action of the ten-eleven translocation (TET) (TET1, 2 and 3) enzymes [5]. These enzymes promote DNA demethylation by converting 5mC to 5-hydroxymethylcytosine (5hmC), which they further oxidize into 5-formylcytosine and 5-carboxylcytosine. The growth arrest and DNA-damage-inducible protein 45A (GADD45A) enzyme could also contribute to demethylation [6], especially in autoimmune diseases [7, 8].

Global DNA hypomethylation has been found in several inflammatory and autoimmune diseases where it entails aberrant expression of genes and ribosomal RNAs probably implicated in their pathology [9, 10]. Other changes associated with DNA hypomethylation as genome instability and mutations, or use of cryptic promoters have not been described in the autoimmune diseases. In RA, DNA hypomethylation has been thoroughly demonstrated in fibroblast-like synovial (FLS) cells [11–14]. Hypomethylation at specific CpG sites in FLS is associated with overexpression of genes that are keys for the disease process. The consequences of DNA hypomethylation in FLS have been highlighted by the activated phenotype that normal FLS acquire after drug (5-azacytidine)-induced demethylation [11]. However, many aspects of DNA methylation in RA remain incompletely explored or are controversial. For example, there are conflicting reports regarding changes in DNA methylation of blood cells [15, 16], or the expression of methyltransferase enzymes [11, 13, 16]. Also, we do not know the methylation status of other important players in RA beyond FLS and blood T cells, the only cell populations studied to date [11–14, 17]. In addition, all the previous studies have analyzed samples from established RA patients and could reflect effects of treatment or of disease evolution. In this respect, there is a report indicating that methotrexate (MTX) reverts DNA hypomethylation in inflammatory arthritis [18], in spite of MTX inhibition of SAM synthesis [19, 20]. Finally, some previous studies have evaluated global DNA methylation with techniques that are insensitive and unable to distinguish 5mC from 5hmC, which has different functional implications. These design and technical issues could have contributed to some of the previous discordant results. Therefore, we aimed to address some of these questions with disease-modifying anti-rheumatic drug (DMARD)-naïve early RA patients, before and after receiving MTX and using sensitive and accurate technology [21, 22].

## Methods

### Patients and controls

New consecutive patients arriving to the Rheumatology Unit and fulfilling the 2010 American College of Rheumatology/European League Against Rheumatism (ACR/EULAR) classification criteria for RA were included from April 2011 to February 2012 [23]. All had clinical symptoms of less than 2 years of evolution compatible with their classification as early RA. Clinical data and blood samples were taken before and 1 month after starting treatment with MTX. Clinical follow-up was done with complete independence from this study. Gender and age-matched healthy controls were recruited at the same time. All patients and controls were of Caucasian Spanish ancestry. The Ethics Committee for Clinical Research of Galicia approved this study, and written informed consent was obtained from all participants.

### Blood cell subpopulations

Double gradient separation by centrifugation was used to isolate granulocytes and mononuclear cells with Histopaque®-1077 and Histopaque®-1119 (Sigma-Aldrich, St Louis, MO, USA) from 20 mL of EDTA anti-coagulated blood as described [24]. Mononuclear cells forming the buffy coat over the 1077 layer were fractionated by immune-magnetic positive selection in four subpopulations CD56+ (natural killer (NK) cells), CD14+ (monocytes), CD19+ (B lymphocytes) and CD3+ (T lymphocytes). The MACS® system (Miltenyi Biotek Bergisch Gladbach, Germany) was used for NK cells and BD IMag™ cell separation system (BD Biosciences, San Jose, CA, USA) for the other subpopulations. Purity of the isolated cells was controlled by fluorescence-activated cell sorting analysis on a FACScan™ cytometer with CellQuest Pro Software (BD Biosciences) as double-stained cells with anti-CD45 PE and subpopulation-specific antibodies labeled with FITC (anti-CD14, CD19, CD3 for the populations purified with these antibodies and anti-CD15 for granulocytes) and as CD56^+^ and CD3^−^ cells for the NK cells. All antibodies were from BD Biosciences. Purity of isolated cell subpopulations ranged from 90 to 99 %. Purified cells were processed with the illustra™ triplePrep Kit (GE Healthcare. Little Chalfont, UK) according to the manufacturer’s instructions to obtain genomic DNA and total RNA.

### Global DNA methylation

5mC relative levels were quantified following enzymatic hydrolysis of genomic DNA as described [21]. Three standards were included in each analysis. They were identical DNA sequences except for the inclusion of 100 % unmodified cytosines, 100 % 5mC or 100 % 5hmC, respectively (Zymo Research, Irvine, CA, USA). Samples and standards, 0.1–1 μg, were hydrolyzed to their component nucleosides before analysis by incubation with DNA Degradase Plus (Zymo Research) for 2 h at 37 °C. Separation and quantification of the nucleosides was done by high-performance liquid chromatography-electrospray ionization-tandem mass spectrometry-selected reaction monitoring (HPLC-ESI-MS/MS-SRM) in the API 4000 LC/MS/MS System (AB Sciex, Framingham, MA, USA) including a ZORBAX Eclipse XDB-C18 column (Agilent Technologies, Santa Clara, CA, USA) and a triple quadrupole mass spectrometer. This system allows the accurate and sensitive differentiation of 5mC from the four deoxyribonucleosides, the four ribonucleosides and 5hmC [21]. Quantification was expressed as the ratio of 5mC to total cytosine (5mC/5mC+C).

### DNA methyltranferase expression analysis

Total RNA from each cell subpopulation was immediately reverse-transcribed with avian myeloblastosis virus reverse transcriptase (Promega, Madison, WI, USA) and random primers. Gene expression of *DNMT1*, *DNMT3A*, *DNMT3B*, *TET1*, *TET2*, *TET3* and *GADD45A* was quantified with quantitative polymerase chain reaction (qPCR). Primers were designed with the primer Express 3.0 software (Applied Biosystems, Foster City, CA, USA) (Table S1 in Additional file 1). Five housekeeping genes were initially tested (*RPL13A*, *GAPDH*, *B2M*, *18S* and *TBP*) as reported [25]. Those showing stable expression (*TBP* and *18S* for all cell subpopulations except granulocytes, where only *TBP* showed stable levels) in qBase [26] were used as reference. Quantifications were performed in triplicate 10 μl reactions containing 1 μl cDNA, 5 μl RT^2^ SYBR Green qPCR Mastermix (Qiagen, Venlo, The Netherlands), and 250 nM of each primer in a Rotor-Gene™ 6000 (Corbett Life Science, Venlo, The Netherlands) thermocycler with initial activation at 95 °C for 10 minutes, followed by a two-step program of 95 °C for 15 seconds and 60 °C for 60 seconds for 45 cycles (gain = 8). Standards were included in each run for inter-run calibration. Specificity of the PCR reactions was confirmed by melting curve analysis of the products as well as by size verification by DNA electrophoresis. Transformed expression data were analyzed with qBase after adjusting for amplification efficiency of each transcript [26].

### Statistical analysis

Data analysis was performed with Statistica 7.0 (StatSoft, Tulsa, OK, USA). Non-normality of the variables was corrected with logarithm or power transformations. Differences between patients and controls were evaluated using main effects analysis of variance with covariates (ANCOVA). Covariates were sex and age. Within-patient comparisons, before and after MTX treatment, were done with paired-samples *t* tests.

## Results

### Characteristics of patients, controls and the isolated blood cell subpopulations

A total of 19 consecutive patients with RA starting MTX treatment were recruited (Table 1). The fraction of men (73.7 %) was higher than the typical of patients with RA. Symptoms had started a median of 6.0 months before MTX treatment with only three patients surpassing the year since the first symptoms and none with more than 20 months of evolution. These patients had not received DMARDs before starting MTX. All had already been treated with low-dose methylprednisolone (4–10 mg/day) for a median of 23 days before starting MTX (interquartile range (IQR) 12–33.5 days). Activity of RA was moderate in most patients with mean disease activity score in 28 joints (DAS28) of 4.6. Only two of the patients showed erosions on radiographic exploration in spite of the presence of anti-citrullinated peptide antibodies (ACPA) or rheumatoid factor (RF) positivity in more than half of them (63.2 % seropositive). The initial dose of MTX was 10 mg/week except for one patient who received 15 mg/week. A second sample from each patient was taken 1 month after starting MTX treatment to assess the effect on DNA methylation and expression of *DNMT*s, *TET*s and *GADD45A*. All patients were in MTX monotherapy at that time and without change in MTX or methylprednisolone doses. Although 1 month is too early for assessing response to MTX, a decrease of RA activity was already evident in most patients (Table 1). Response to MTX was assessed at 6-month follow-up. At that time, 15 patients remained in monotherapy with MTX, 12 of them showed good response according to the EULAR criteria [27], one showed a moderate response and two were non-responders. Of the four patients not remaining on MTX monotherapy at 6 months, two were on a different DMARD due to inefficacy of MTX and two had interrupted MTX due to adverse effects. A total of 17 healthy controls matched for age and sex were recruited and studied.

**Table 1:** Characteristics of the patients with early RA and of the healthy controls included in the study

	RA patients	Healthy controls
Number	19	17
Female, (%)	5 (26.3)	6 (35.3)
Age at diagnosis, median (IQR)	61.6 (51–65)	58 (39–64)
Symptoms to MTX, median months (IQR)	6.0 (2.2–7.3)	
RF (%)	11 (57.9)	
ACPA (%)	11 (57.9)	
Erosive arthritis (%)	2 (10.5)	
Smoking (%)	8 (42.1)	
Methylprednisolone, median mg/day (IQR)	4 (4–8)	
Metothrexate, median mg/week (IQR)	10 (10–10)	
CRP (mg/L), median (IQR)		
Baseline	15.3 (3.9–23.2)	
1 month	5.2 (1.2–9.4)	
ESR, median (IQR)		
Baseline	28.0 (6.0–38.0)	
1 month	14.0 (6.5–24.0)	
DAS28, mean ± SD		
Baseline	4.6 ± 1.6	
1 month	3.2 ± 1.5	

*RA* rheumatoid arthritis, *IQR* interquartile range, *MTX* methotrexate, *RF* rheumatoid factor, *ACPA* anti-citrullinated peptide antibody, *CRP* C-reactive protein, *ESR* erythrocyte sedimentation rate, *DAS28* disease activity factor in 28 joints, *SD* standard deviation

Three subpopulations of blood cells monocytes, B and T lymphocytes were isolated from the 19 patients and 17 healthy controls. Granulocytes and NK cells were also isolated in the first eight patients and eight controls but not pursued further because no differences were observed. The results from these two blood subpopulations will not be presented in detail. Purity of the isolated subpopulations ranged from 90 to 99 %.

### Differences in global DNA methylation between patients with RA and controls

**Fig. 1:**
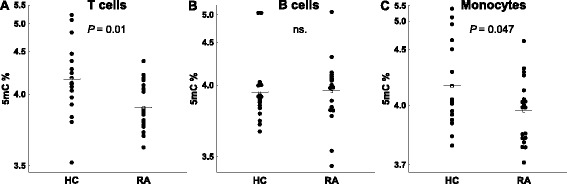
Differences in global DNA methylation between healthy controls (HC) and early rheumatoid arthritis (RA) patients. The percentage of 5-methylcytosine (5mC) over the total content of cytosine in total DNA of **a** T cells, **b** B cells, and **c** monocytes is shown. *Each dot* represents a subject. *Horizontal bars with an empty square* are means. Differences in (**a**) and (**c**) were significant, *P* < 0.05

**Fig. 2:**
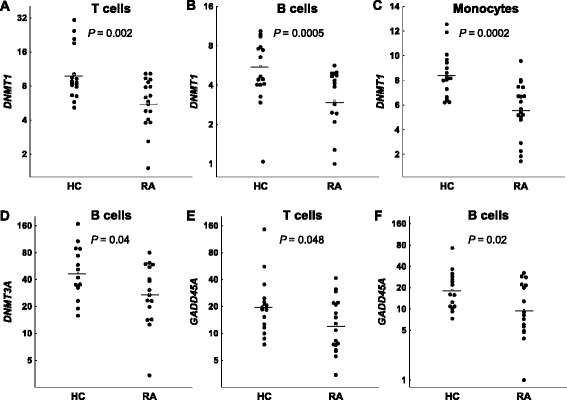
Expression of methylation enzymes in blood cells of healthy controls (HC) and early rheumatoid arthritis (RA) patients. Normalized relative expression of *DNMT1* obtained by quantitative polymerase chain reaction (qPCR) in **a** T cells, **b** B cells, and **c** monocytes, *DNMT3A* in B cells (**d**) and of *GADD45A* in T cells (**e**) and B cells (**f**) is shown. Other conventions are as in Fig. 1. All these comparisons were significant. No comparison in other cells subpopulation and none of the *DNMT3B* analyses were significant

**Fig. 3:**
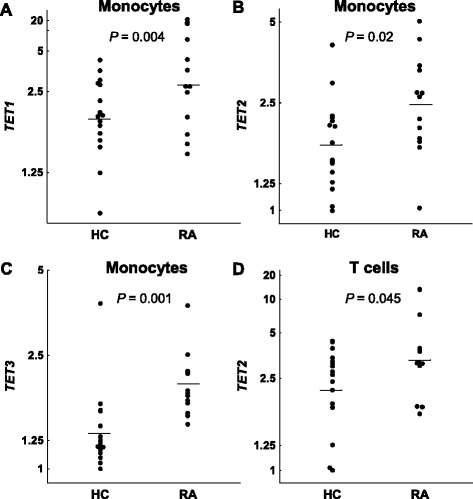
Differences between healthy controls (HC) and early rheumatoid arthritis (RA) patients in ten-eleven translocation (TET) enzymes expression. Normalized relative expression of **a** *TET1*, **b** *TET2*, and **c** *TET3* in monocytes; and of **d** *TET2* in T cells. Conventions are as in Fig. 1. All differences were significant at *P* < 0.05

Levels of global 5mC were similar to those previously reported [21, 22]. Comparison of the global 5mC level between patients and controls was done with the samples of RA patients before MTX treatment. Main effects ANCOVA with sex and age as covariates was used for these comparisons. T lymphocytes showed significant DNA hypomethylation in early RA patients compared with healthy subjects (Fig. 1a; mean = 3.89 %, 95 % confidence interval (CI) = 3.80–3.99 vs*.* 4.15 %, 95 % CI = 3.96–4.38, respectively, *P* = 0.011). The multivariate analysis also showed significant DNA hypomethylation in the monocytes of RA patients (Fig. 1c; mean = 3.96 %, 95 % CI = 3.87–4.07 vs*.* 4.13, 95 % CI = 3.98–4.33, respectively, *P* = 0.047). Although significant, these differences were small. No significant differences were detected in any of the other blood cell subpopulations: B lymphocytes, NK cells and polymorphonuclear (PMN) cells (Fig. 1b and not shown). No significant correlation between the total methylprednisolone dose received until blood drawing and levels of 5mC in any of the five blood subpopulations were observed in RA patients (not shown). In the same assay, 5hmC was also determined without any significant difference between patients and healthy controls, showing very low levels in all cell subpopulations (around 0.02 % of total cytosine) as is characteristic of most adult tissues [21, 28]. *DNMT1*, *DNMT3A*, *DNMT3B*, *TET1*, *TET2*, *TET3* and *GADD45A* relative expression was determined by qPCR analysis in each of the blood cell subpopulations. A significant decrease in patients with RA was observed for some enzymes: *DNMT1* expression was decreased in T cells (Fig. 2a; mean = 5.6, 95 % CI = 4.3–7.2 vs. 10.0, 95 % CI = 7.7–13.1; *P* = 0.0022), B cells (Fig. 2b; mean = 3.4, 95 % CI = 2.6–4.3 vs. 6.0, 95 % CI = 4.6–7.4; *P* = 0.00046) and monocytes (Fig. 2c; mean = 5.6, 95 % CI = 4.5–6.7 vs. 8.4, 95 % CI = 7.4–9.4; *P* = 0.00020); *DNMT3A* was decreased, although less markedly, in B cells (Fig. 2d; mean = 27.5, 95 % CI = 17.9–42.2 vs. 46.9, 95 % CI = 32.4–67.9; *P* = 0.044); no difference was found in the expression of *DNMT3B* in any of the blood cell subpopulations (not shown); and *GADD45A* expression was also moderately reduced in T cells (Fig. 2e; mean = 12.2, 95 % CI = 8.7–17.3 vs. 20.0, 95 % CI = 13.9–28.8; *P* = 0.048) and in B cells (Fig. 2f; mean = 9.6, 95 % CI = 5.8–45.9 vs. 18.3, 95 % CI = 12.9–25.9; *P* = 0.021). On the contrary, the relative expression of *TET* enzymes was increased in some cell populations. Monocytes showed the largest increases, with the three *TET* enzyme genes showing higher relative levels of expression in RA patients than in controls. *TET1* and *TET3* showed the most marked difference between RA patients and controls (*TET1*, Fig. 3a; mean = 2.9, 95 % CI = 2.1–4.8 vs. 1.9, 95 % CI = 1.6–2.3; *P* = 0.0044; and *TET3*, Fig. 3c; mean = 2.0, 95 % CI = 1.7–2.3 vs. 1.35, 95 % CI = 1.15–1.6; *P* = 0.0014), but also *TET2* (Fig. 3b; mean = 2.5, 95 % CI = 1.9–3.2 vs. 1.8, 95 % CI = 1.5–2.2; *P* = 0.019) was increased in monocytes of the patients with RA. *TET2* was also modestly increased in the T cells of RA patients in relation with the healthy controls (Fig. 3d; mean = 3.4, 95 % CI = 2.4–5.2 vs. 2.2, 95 % CI = 1.8–3.0; *P* = 0.045). No other differences were detected.

### Increase of global DNA methylation after MTX treatment

**Fig. 4:**
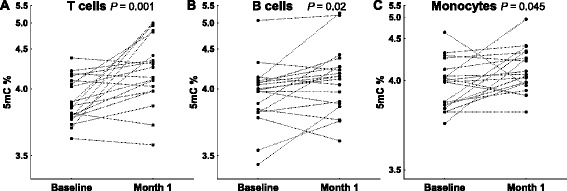
Increased global DNA methylation in rheumatoid arthritis (RA) patients after 1 month on methotrexate (MTX). The percentage of 5-methylcytosine (5mC) over the total content of cytosine in total DNA of **a** T cells, **b** B cells, and **c** monocytes is shown. *Each pair of dots joined by dashed lines* represents a subject before and after MTX. All changes were significant with *P* < 0.05

Global DNA methylation was analyzed 1 month after starting treatment with MTX. Comparison of the percentage of 5mC in each of the five blood cell subpopulations with *t* tests for dependent variables showed significant increases after treatment in three of them: T cells that showed the most significant increase in global DNA 5mC (Fig. 4a; mean = 4.21 %, 95 % CI = 4.03–4.43 after 1 month of treatment; *P* = 0.0014), B cells (Fig. 4b; 4.09 %, 95 % CI = 3.94–4.28 after treatment vs. 3.94 %, 95 % CI = 3.82–4.09 before MTX; *P* = 0.018) and monocytes that showed a borderline increase (Fig. 4c; 4.09 %; 95 % CI = 4.00–4.21 after 1 month of treatment; *P* = 0.045). However, some patients did not follow this trend to recovery (Fig. 4). No differences in 5mC were detected in NK or PMN cells between baseline and after 1 month with MTX. No detectable variation in global 5hmC levels was observed (data not shown).

**Fig. 5:**
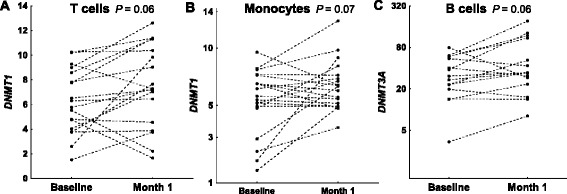
Largest changes in expression of methylation enzymes after 1 month on methotrexate (MTX). Normalized relative expression of **a** *DNMT1* in T cells, and **b** in monocytes, and **c** *DNMT3A* in B cells, before and after 1 month on MTX is shown. Figure conventions are as in Fig. 4. The three changes showed *P* < 0.1, but none was significant

We have also compared expression of *DNMT1*, *DNMT3A*, *DNMT3B*, *GADD45A*, *TET1*, *TET2* and *TET3* in the five cell subpopulations after MTX treatment with their baseline levels. No clear changes were observed (Fig. 5). Only *DNMT1* showed a trend to increased levels after treatment in T cells and monocytes that was near significant (*P* = 0.06 and 0.07, respectively). A not significant trend to an increase of *DNMT3A* in B cells (*P* = 0.06) was also observed. No similar trend was found for any of the other enzymes. On the contrary, the differences with healthy controls that were present before treatment were still present after 1 month on MTX except for *TET2* in T cells that was no longer significantly different. In particular, the TET enzyme expression in monocytes showed the same level of difference with healthy controls than before treatment (*TET1 P*_*before*_ = 0.037, *P*_*after*_ = 0.039; *TET2 P*_*before*_ = 0.019, *P*_*after*_ = 0.013; and *TET3 P*_*before*_ = 0.0014, *P*_*after*_ = 0.0033), as well as the expression of *GADD45A* in T and B cells (T cells *P*_*before*_ = 0.048, *P*_*after*_ = 0.049; B cells *P*_*before*_ = 0.021, *P*_*after*_ = 0.041). Therefore, TET enzyme gene expression in monocytes and GADD45A in T and B cells were not significantly modified by this treatment.

## Discussion

Our results support the involvement of decreased DNA methylation in RA and its reversal in response to MTX. They also widen the spectrum of changes in methylation and in the enzymes regulating this process in RA, as well as provide cell-specific results. In particular, this is the first time that changes in B cells and in monocytes have been described and that the main methylation-related enzymes have been studied in the blood of RA patients. The results support the implication of DNA hypomethylation in RA and highlight the interaction between MTX and DNA methylation that could contribute to its therapeutic efficacy in RA.

A couple of previous studies have analyzed whole peripheral blood mononuclear cells (PBMCs) with discordant results [15, 16]. One of these studies found global DNA hypomethylation in PBMCs with an enzyme-linked immunosorbent assay (ELISA) kit of questionable accuracy given the low percentage of 5mC (1.22 % in controls) [16] in comparison with our results, other results in RA T cells [17], and in other cells and tissues [21, 22]. The second study did not find differences in DNA methylation between PBMCs of RA discordant monozygotic twins at 1505 CpG sites analyzed by bead array [15]. However, these sites were not randomly distributed and their representativeness of global DNA methylation is uncertain, and could be insensitive to detect the small difference we have observed. The most specific study to date has analyzed PBMCs in two subsets, T cells and non-T cells, with a HPLC method [17]. The T cells showed global DNA hypomethylation, whereas the non-T cells did not. These results are in agreement with our observations.

Meaning of global DNA hypomethylation in autoimmune diseases has been best studied in systemic lupus erythematosus (SLE) CD4 T cells. These cells show global and site-specific hypomethylation that is associated with increased expression of immune response genes, many of them in the interferon (INF) signaling pathway, overexpression of ribosomal RNA, modifications of imprinting, and reactivation of endogenous retrovirus [8–10, 29]. All these changes could contribute to the breakdown of immune tolerance and to chronic inflammation. Support of this causal role has been provided by the drug-induced SLE that follows treatment with hydralazine or procainamide, which decrease DNA methylation, and by animal studies [8–10]. Although some of the above-mentioned changes could be SLE-specific, it is likely that DNA hypomethylation in RA T cells has a similar role. In this regard, there is already evidence showing that specific CpG sites at *TNFSF5* are similarly hypomethylated in T cells of SLE and RA [30]. In addition, a recent large study in RA has found evidence for methylation changes in PBMCs as possible mediators in genetic susceptibility [3].

As already mentioned, global DNA methylation has not been previously analyzed in monocytes of RA patients. In SLE, monocytes share several hypomethylated sites with CD4 T cells, although they were more numerous and prominently hypomethylated in T cells [29]. In RA monocytes, the methylation level at a CpG site in the *IL6* promoter that was hypomethylated in RA PBMCs was inversely correlated with interleukin (IL)-6 lipopolysaccharide (LPS)-induced expression [31]. It can only be presumed that changes in methylation as this one could also contribute to the disease process.

*DNMT1* was the most markedly and consistently decreased DNA methyltransferase in our study. This result is in contrast with a previous report that found increased *DNMT1* expression in PBMCs of RA patients [16], but it is in agreement with multiple observations in other autoimmune diseases [8, 9, 15]. In addition, impaired DNMT1 function has a causal role in global DNA hypomethylation and autoimmunity as demonstrated by drug-induced SLE [9]. In RA, studies of DNMT1 have been much more limited and restricted to FLS in culture. They show comparable levels to the observed in osteoarthritis (OA) FLS, but *DNMT1* expression is markedly downregulated by incubation with small amounts of inflammatory cytokines [11, 13]. These previous reports suggest possible mechanisms for the decreased *DNMT1* expression and indicate that downregulation of DNMT1 is very likely a major factor in DNA hypomethylation. However, correlation between *DNMT1* levels and DNA hypomethylation was not present in B cells in our study. The lack of hypomethylation in B cells was especially remarkable because they were the only cell type showing decreased expression of *DNMT1* and *DNMT3A*. No reduction of *DNMT3A* levels has been previously described in RA FLS or in SLE T cells [13, 32–34], which are the two cell types that have been studied. In turn, no differences in DNMT3B have been described in agreement with our negative results regarding this enzyme [13, 32–34].

In addition to the changes in the enzymes that methylate DNA, our study is the first addressing the DNA demethylases in an autoimmune disease. Expression of the genes for the three TET enzymes were increased in the monocytes of RA patients, and *TET2* was also increased in T cells. These results could contribute together with the decrease in *DNMT1* to the global hypomethylation observed in the patients. Of possible relevance for RA is that the *TET3* locus has been associated with SLE susceptibility in Asians [35], and that *TET1* regulates transcription and processing of *IL1β* and other pro-inflammatory genes in experiments with cell lines [36]. Previously, GADD45A was the main known active DNA demethylase [6], however this role seems to correspond to the TET enzymes [5]. However, interest in GADD45A has remained in the autoimmune diseases because it is overexpressed in CD4 T cells of SLE patients, correlating with global and site-specific DNA hypomethylation in these cells [7, 8]. In contrast, *GADD45A* expression was diminished in our analysis of RA patients, both in T and B cells. The observed decrease is not congruent with global DNA hypomethylation in T cells given its proposed demethylating function, but GADD45A has other functions and one of them could have a role here: its role as inhibitor of T cell receptor (TCR) signaling [37].

Several of the differences we observed in RA patients were reverted after 1 month of MTX treatment. First, global DNA hypomethylation in T cells, B cells and monocytes was reverted in most patients. These results are reminiscent of a smaller study including patients with inflammatory arthritis (RA and psoriatic arthritis (PsA)) on long-term treatment with MTX [18]. This outcome is contrary to the expected given the MTX suppression of SAM [38], which is the major source of methyl groups for DNA methylation. However, control of inflammation by MTX could lead to reduced cell proliferation, which is a source of SAM consumption through increased recycling of polyamines [39], and to reversal of *DNMT1* and *DNMT3A* expression given their sensitivity to pro-inflammatory cytokines [11, 13]. The two mechanisms could contribute to the recovery of DNA methylation.

## Conclusions

Our results support the implication of DNA methylation in RA and in its response to MTX treatment. They also widen the spectrum of changes and the types of cells that are affected by them: global DNA hypomethylation in T cells and monocytes associated with a lower expression of *DNMT1* and increased expression of the three TET enzymes in monocytes and *TET2* in T cells, together with decreased *DNMT1* and *DNMT3A* expression in B cells. Several of these changes were reverted after MTX treatment, most remarkably the DNA global hypomethylation. The differences and changes found here in peripheral blood could reflect important mechanisms both in disease evolution and in its control by MTX and suggest multiple new areas of future research.

## Additional file

Additional file 1: Table S1. Primers used for assessing the expression level of methylation-relevant enzymes by qPCR. (DOC 36 kb)

## Abbreviations

5hmC: 5-hydroxymethylcytosine
5mC: 5-methylcytosine
ACPA: anti-citrullinated peptide antibody
ACR: American College of Rheumatology
ANCOVA: analysis of variance with covariates
CI: confidence interval
CpG: cytosine-phosphate-guanine dinucleotide
DAS28: disease activity score in 28 joints
DMARD: disease-modifying anti-rheumatic drug
DNMT: DNA methyltrasnferase
EULAR: The European League Against Rheumatism
FLS: fibroblast-like synoviocyte
GADD45A: growth arrest and DNA-damage-inducible protein 45 alpha
HPLC-ESI-MS/MS-SRM: high-performance liquid chromatography-electrospray ionization-tandem mass spectrometry-selected reaction monitoring
IQR: interquartile range
MTX: methotrexate
NK: natural killer
PBMCs: peripheral blood mononuclear cells
PMN: polymorphonuclear
qPCR: quantitative polymerase chain reaction
RA: rheumatoid arthritis
RF: rheumatoid factor
SAM: S-adenosyl-L-methionine
SLE: systemic lupus erythematosus
TET: ten-eleven translocation
