# Supplementary material for: Assessment of global DNA methylation in peripheral blood cell subpopulations of early rheumatoid arthritis before and after methotrexate
Source: Arthritis Res Ther. 2015 Aug 29;17(1):233. doi: 10.1186/s13075-015-0748-5 (PMC4556005; doi:10.1186/s13075-015-0748-5)
Supplement: Additional file 1: Table S1. — Primers used for assessing the expression level of methylation-relevant enzymes by qPCR. (DOC 36 kb) [file 13075_2015_748_MOESM1_ESM.doc]

Table S1: Primers used for assessing the expression level of methylation relevant enzymes by qPCR

| Gene | Accesion No. | Forward primer | Reverse primer | Amplicon size (bp) |
| --- | --- | --- | --- | --- |
| DNMT1 | NM_001130823.1 | caggcccaatgagactgaca | gtgggtgttctcaggcctgtag | 64 |
| DNMT3A | NM_175629.1 | cctactacatcagcaagcgcaa | ttccacagcattcattcctgc | 101 |
| DNMT3B | NM_006892.3 | ccatgaaggttggcgacaa | tggcatcaatcatcactggatt | 69 |
| GADD45a | NM_001924.3 | tgctcagcaaagccctgagt | gcaggcacaacaccacgtta | 99 |
| TBP | NM_003194 | tgcacaggagccaagagtgaa | cacatcacagctccccacca | 132 |
| 18S | NM_022551 | atccctgaaaagttccagca | ccctcttggtgaggtcaatg | 186 |
| GAPDH | NM_002046 | gagccacatcgctcagacac | catgtagttgaggtcaatgaagg | 150 |
| RPL13A1 | NM_012423 | aaaaagcggatggtggttc | cttccggtagtggatcttgg | 168 |
| β2M | NM_004048 | atgagtatgcctgccgtgtga | ggcatcttcaaacctccatg | 101 |
